# Dissolved Oxygen Sensor in Animal-Borne Instruments: An Innovation for Monitoring the Health of Oceans and Investigating the Functioning of Marine Ecosystems

**DOI:** 10.1371/journal.pone.0132681

**Published:** 2015-07-22

**Authors:** Frederic Bailleul, Jade Vacquie-Garcia, Christophe Guinet

**Affiliations:** 1 South Australian Research and Development Institute (Aquatic Sciences), 2 Hamra Avenue, West Beach, Adelaide, SA, 5024, Australia; 2 Centre d’Etudes Biologiques de Chizé, UMR 7372 (CNRS/Universite de la Rochelle), 79360, Villiers en Bois, France; Musee National d'Histoire Naturelle, FRANCE

## Abstract

The current decline in dissolved oxygen concentration within the oceans is a sensitive indicator of the effect of climate change on marine environment. However the impact of its declining on marine life and ecosystems’ health is still quite unclear because of the difficulty in obtaining *in situ* data, especially in remote areas, like the Southern Ocean (SO). Southern elephant seals (*Mirounga leonina*) proved to be a relevant alternative to the traditional oceanographic platforms to measure physical and biogeochemical structure of oceanic regions rarely observed. In this study, we use a new stage of development in biologging technology to draw a picture of dissolved oxygen concentration in the SO. We present the first results obtained from a dissolved oxygen sensor added to Argos CTD-SRDL tags and deployed on 5 female elephant seals at Kerguelen. From October 2010 and October 2011, 742 oxygen profiles associated with temperature and salinity measurements were recorded. Whether a part of the data must be considered cautiously, especially because of offsets and temporal drifts of the sensors, the range of values recorded was consistent with a concomitant survey conducted from a research vessel (Keops-2 project). Once again, elephant seals reinforced the relationship between marine ecology and oceanography, delivering essential information about the water masses properties and the biological status of the Southern Ocean. But more than the presentation of a new stage of development in animal-borne instrumentation, this pilot study opens a new field of investigation in marine ecology and could be enlarged in a near future to other key marine predators, especially large fish species like swordfish, tuna or sharks, for which dissolved oxygen is expected to play a crucial role in distribution and behaviour.

## Introduction

For the last decades, several studies based on measurements *in situ* and modelling have clearly noted a decreasing trend in the concentration of dissolved oxygen in the oceans, for which climate change was identified as a critical factor [1]. First, the solubility of oxygen drops as the ocean waters get warmer. Second, warmer ocean waters are more stable, thereby slowing down the ocean circulation system [2] and reducing the vertical mixing processes between water layers. The result is less oxygen transported from the oxygen-rich surface layer (in contact with air) into the deep ocean, where zones depleted in dissolved O_2_ (oxygen minimum zones–OMZ) are expanding [3, 4]. In addition, the slowing down of the ocean’s circulation system also results in a reduced supply of nutrients from the deep layers into the ocean surface. The low availability of nutrients in the surface layers causes the decline of phytoplankton biomass and diversity (i.e. oxygen producers) [5] and hence, the level of oxygen at surface is bound to decline further. Influenced both by physical and dynamical characteristics of water masses and biological production, dissolved O_2_ proves to be a sensitive indicator of changes in properties of marine environment [6].

However, although the critical role of dissolved O_2_ for indicating changes in the oceans clearly appears, the future impact of its declining on marine life and ecosystems’ health is still quite unclear. A decreasing in dissolved oxygen concentration could affect the biological activity of micro-organisms at depth and hence, generate perturbation in the functioning of ecosystems and accelerate changes in biogeochemical cycling [7]. Moreover, many marine taxa are unable to abide low oxygen concentration and a decline in oxygen should lead to habitat compression for hypoxia-intolerant species, with eventual loss of biodiversity [4]. In every cases, clearly addressing physical or biological issues related to dissolved oxygen in the marine ecosystems requires a large dataset of *in situ* measurements.

Up to few years ago, accurate oxygen measurements were performed from infrequent and geographically-limited research vessel surveys. Recent improvements in sensor technology have nevertheless enabled a number of new bio-geochemical sensor packages to be deployed on new autonomous measurement platforms, such as profiling floats, gliders or moorings. As an example, over 400 Argo floats, dispersed in the global ocean, record dissolved oxygen besides temperature and salinity (source: IMOS, Integrated Marine Observation System). However, despite these significant advances, the physical and biogeochemical properties of some regions remain challenging to investigate, using such observing systems. Because of its remoteness, regular harsh weather conditions and seasonal presence of sea ice, the Southern Ocean (SO) remains largely under-sampled by the conventional instruments. But more than just a sampling issue, overcoming the lack of biogeochemical data in the SO is even more critical, as this region displays the largest change in the air-sea exchange of oxygen with climate change [8].

In this context, the development of new ways to sample the SO is very useful. The development of original bio-logging devices, deployed on southern elephant seals (SES) since 2003/2004, led to a significant increase in samples of vertical profiles of temperature, salinity and fluorescence in the remote SO [9]. This methodology was very efficient delivering near-real time physical and biogeochemical oceanographic data as well as new behavioural information on free ranging seals, which explore large areas in the SO and dive continuously at great depths.

Here we promote a new stage of development of the animal-borne instruments with the addition of a sensor measuring dissolved O_2_ in the Argos CTD-SRDL tags (Argos-linked Conductivity–Temperature–Depth-Satellite Relayed Data Loggers-SMRU- University of St Andrews Scotland). We present the new measurements obtained from these devices, recently deployed on Kerguelen elephant seals, in the oceanographic and biogeochemical context of the SO. We investigate the quality and accuracy of the measurements using existing data from a concomitant research vessel survey. We discuss how monitoring routinely this parameter on the long term using top predators improves the observational database of dissolved O_2_ in the SO and represents a new opportunity to investigate the relationship between air-breathing marine predators and the other components of marine ecosystems. Finally, we enlarge the discussion on a future way of research focused on the relationship between marine predators and their prey, which remain dependent on dissolved O_2_. This point should lead to a new insight in the role of oxygen in functioning and structuring pelagic ecosystems.

## Materials and Methods

### Ethics statement

The protocol of this study was approved by the ethics committee for experiment on animals of the French Polar Institute (Institut Paul Emile Victor–IPEV) in May 2008. Animals were handled and cared for in total accordance with the guidelines and recommendations of this committee (dirpol@ipev.fr). All tag deployments were performed under anesthesia, and all efforts were made to minimize negative impact on animals.

### Instrumentation

In the last decade, the CTD-SRDLs have been used on a range of marine mammals [10–12] but more intensively on Kerguelen elephant seals each year since 2003 and a comprehensive technical description of these tags can be found in several papers [13, 14]. Briefly, the devices contain a Platform Terminal Transmitter (PTT) transmitting summarized data via the ARGOS satellite system in near real time when the animal is at the surface and a miniaturised CTD (Valeport LTD, Totnes, UK) collecting ocean temperature and conductivity vertical profiles as well as information on depths of dives. The features of the different sensors (pressure transducer, temperature probe and conductivity sensor) such as the range of measurements and the accuracy are available on the SMRU website (http://www.smru.st-and.ac.uk/Instrumentation/CTD).

### Implementation of an oxygen sensor

The optode 4330 F is a compact cylinder (diameter: 36 mm—total length: 86 mm) designed to measure absolute oxygen concentration (Aanderaa Instruments and Precision Sensing GmbH, http://www.aanderaa.com). The measurement is based on the ability of dissolved oxygen to act as a dynamic fluorescence quencher, i.e. which decreases fluorescence intensity. The sensor (range: 0–500 μM, accuracy <8 μM, response time <8 sec) includes an optical part and its own digital signal processor that treats the signal, compensates for the calibration constants and gives out absolute readings of oxygen in micromoles per liter. Moreover, a thermistor measuring temperature is also incorporated in order to correct the measurement for temperature variations, which affects the response of the sensor foil. The oxygen optode was integrated in a classical CTD-SRDL by the SMRU. As Argos messages are restricted in length, the number of oxygen profiles transmitted was limited to 2 or 3 per day, as well as temperature and salinity profiles. For each profile, dissolved oxygen concentration has been sampled from the maximum depth to the surface during the ascent phase of seal’s dives, so that there was always 24 points per profile regardless of the maximum depth (“broken stick algorithm”) [15].

### Deployment of devices

The CTD-Oxy-SRDLs were deployed on five post-breeding females SES in October 2010 (1 individual) and in October 2011 (4 individuals). Animals were captured at Kerguelen Islands (49.35°S / 70.22°E) with a canvas head-bag and anesthetized using a 1:1 combination of Tiletamine and Zolazepam (Zoletil 100) injected intravenously [16, 17].The CTD-Oxy-SRDL tags were glued on the seal’s head using quick-setting epoxy (Araldite AW 2101).

### Analyses

#### Assessment of data quality

Except the Aanderaa factory calibration, it was not possible for logistic issues to perform additional procedures of calibration and *in situ* control for this pilot study. Nevertheless, we compared our measurements with data from an *in situ* survey conducted from a research vessel in October-November 2011 mainly east of Kerguelen Islands (KEOPS2-project: Kerguelen Ocean and Plateau Study 2). More specifically, we compared some Oxygen-Depth profiles recorded by seals to the data recorded by the nearest Keops2 stations in space and time. We also investigated the potential occurrence of temporal drifts for each sensor record using deep data.

#### Apparent Oxygen Utilization

The apparent oxygen utilisation (AOU) is a measure of biologically produced/utilized oxygen and is determined by the difference between the measured dissolved oxygen concentration and the oxygen saturation of a water masse:

$$AOU\;=O_{2sat}\;-\;O_{2meas}$$

While primary production liberates oxygen and increases its concentration, respiration of micro-organisms and degradation of organic matter consumes oxygen and decreases its concentration at depth. Consequently, the lower the AOU of uppermost water layers is, the more important is the primary production.

The oxygen saturation (O_2*sat*_) with respect to temperature and salinity was calculated using formula detailed in Benson & Krause (1984) [18].

#### Remote sensing environmental data from satellite

The oceanographic and biogeochemical context of the study area (encompassing all the tracks) has been drawn using remote sensing sea surface temperatures (SST) and chlorophyll-a concentrations (Chl-a). The study area extended -66°S/-45°S and 50°E/110°E. Mean values for both variables were calculated over the whole study area for the two study periods (Oct 2010-Janv 2011 and Oct 2011-Janv 2012). Mean values of sst and chl-a were then extracted under each seal location. Oceanic fronts, separating main oceanic regions, were located using SST and literature.

SST and Chl-a data were respectively obtained from http://www.ncdc.noaa.gov/oisst and http://disc.sci.gsfc.nasa.gov/giovanni.

## Results

### Data recorded

**Fig 1:**
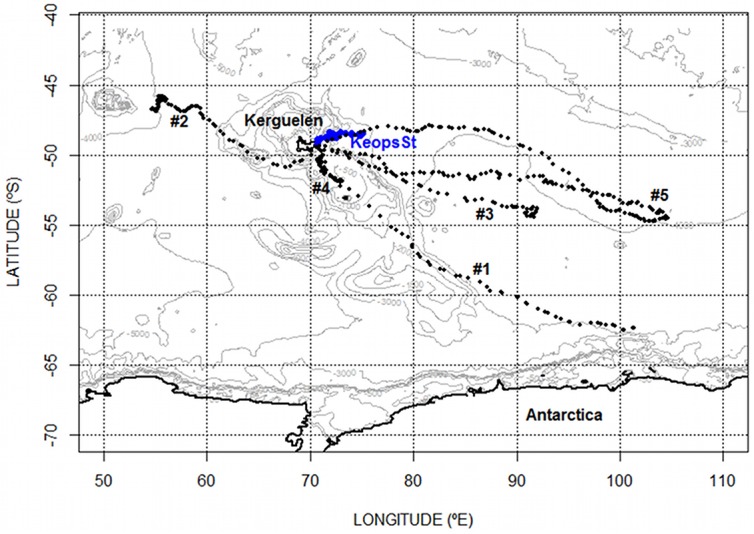
Location of oxygen profiles recorded by Kerguelen elephant seals and the ship survey Keops2. The seal profiles were obtained during the post-breeding foraging trip of 5 females. The individual #1 was equipped in October 2010. The 4 other females (#2 to #5) were equipped in October 2011. Blue dots correspond to the Keops2 stations (Keops St). The Keops survey was conducted in October and November 2011. Isobaths are illustrated in light gray.

During the study periods, a total of 742 vertical oxygen, temperature and salinity profiles were recorded over a total of 389 days, which corresponds to an average of 1.9 ± 0.06 profiles per day per seal. The raw data are attached in S1 Dataset. The number of oxygen seals’ profiles represents 49% of all the oxygen profiles recorded in the same region during the same period. The other profiles having been provided by Argo-oxygen floats (source: http://www.coriolis.eu.org/Data-Services-Products). Moreover many seals’ profiles covered the Kerguelen plateau a zone otherwise largely under-sampled by floats (source: http://www.coriolis.eu.org/Data-Services-Products). Locations of all the seals’ profiles are represented along the track of each individual in the Fig 1, as well as the nearest stations conducted during the Keops2 survey.

### Assessment of data quality

**Fig 2:**
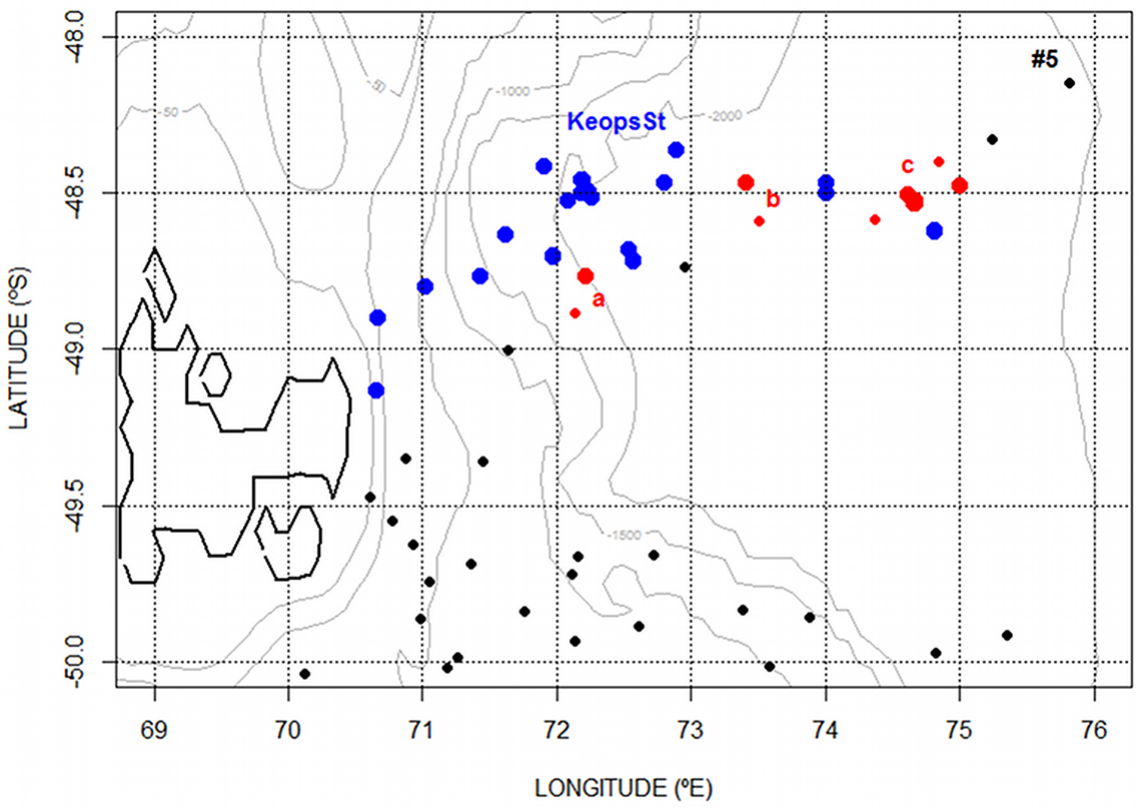
Zoomed location of seal and Keops profiles that have been compared. The selected profiles are in red (large dots = Keops; small dots = Seals). The letters a,b & c correspond to the profiles presented in Fig 3.

**Fig 3:**
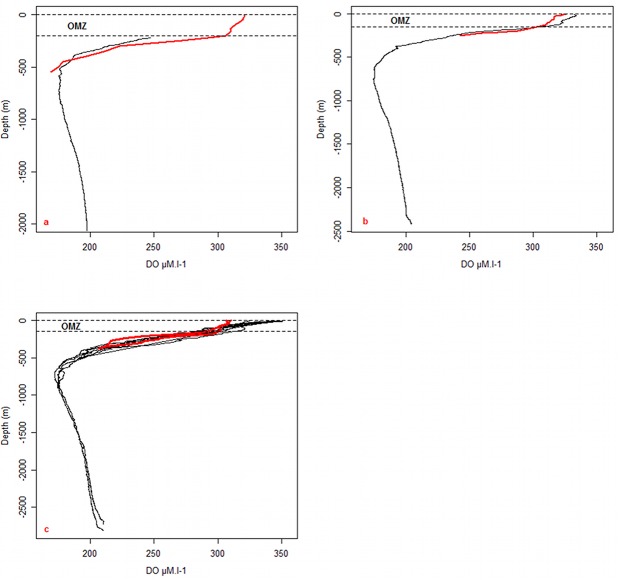
Comparison between the seal (red lines) and the nearest Keops Oxygen-Depth profiles (black lines). A gap between the profiles appears in the Oceanic Mixed Layer (OML). The letters a,b & c correspond to the profiles located in the Fig 2.

**Fig 4:**
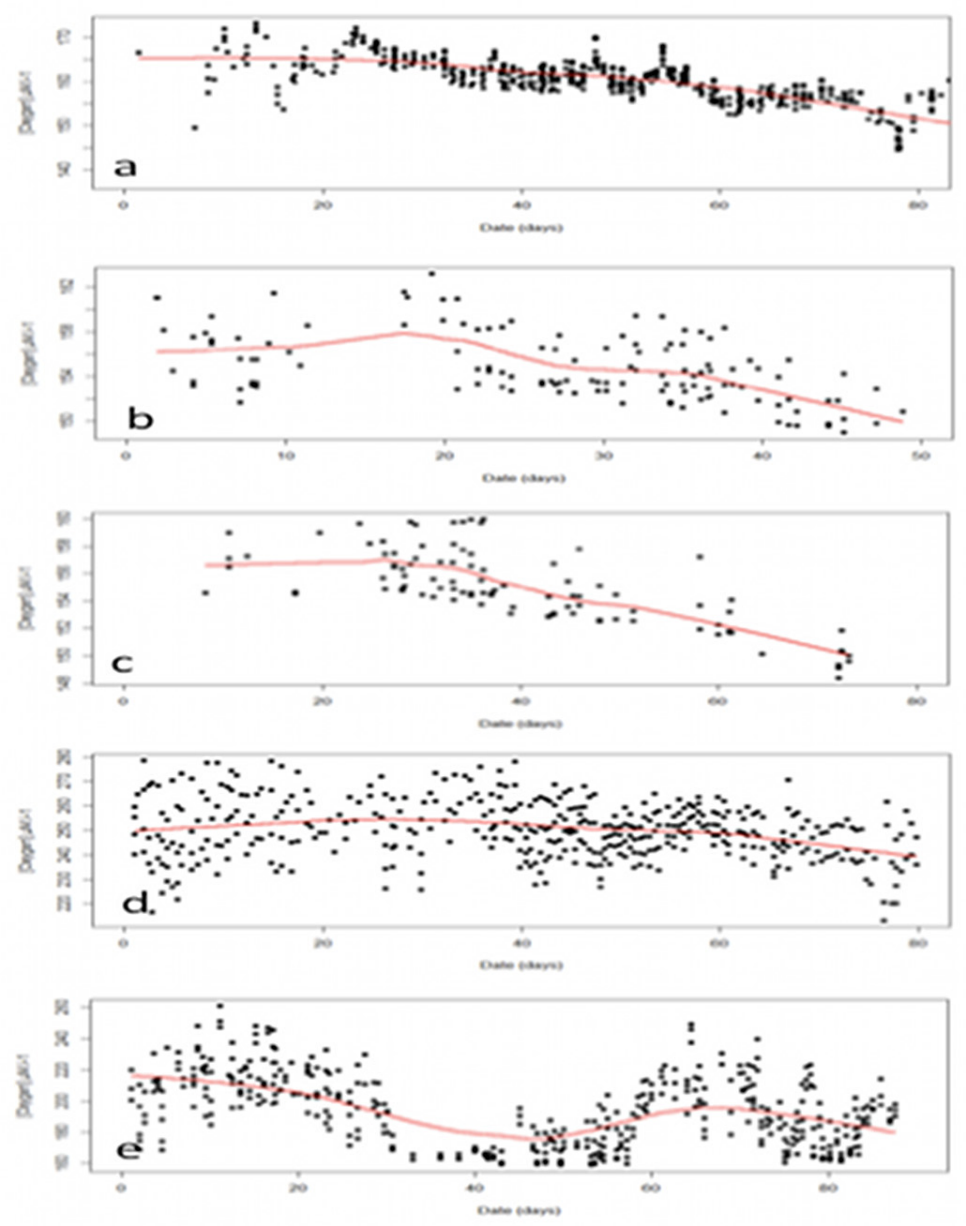
Temporal drift of the seal oxygen sensors. Deep oxygen values (> 400 m) as a function of time showing a constant decrease in oxygen value that start after approximately 30 days at sea. Red lines represent fitting curves.

The values of oxygen concentration measured by seals ranged from 137.22 μM.l^-1^ to 343.52 μM.l^-1^. These values are consistent with those recorded during the ship survey Keops2: 168.92 μM.l^-1^ to 355.98 μM.l^-1^. However, from the comparison between some seal profiles and Keops2 data it should be noted that the maximum values recorded by seals tend to be approximately 10 μM.l^-1^ lower than expected, especially in the oceanic mixed layer, shallower than 150–200 m (Figs 2 and 3). Although the comparison was conducted on very few profiles that did not exactly match in time and space, this gap could be recurrent in the whole seal dataset. Furthermore, the minimum values recorded by seals are about 30 μM.l^-1^ lower than those recorded from the ship. Although, an offset between the different instruments cannot be excluded, such a difference could also be explained by a temporal drift of the oxygen sensor. Such a drift was observed at depth for all but one sensor deployed on seals. The average drift of the oxygen sensors was found to be 0.3 ± 0.1 μM.l^-1^ per day, but only after approximately 30 days at sea. During the first 30 days the sensors appeared to be stable (Fig 4A–4E). In the rest of the study, only the first 30 days of each track (except for the individual #5, for which no drift was observed) will be interpreted.

### Dissolved oxygen in the oceanographic and biogeochemical context of the SO

**Fig 5:**
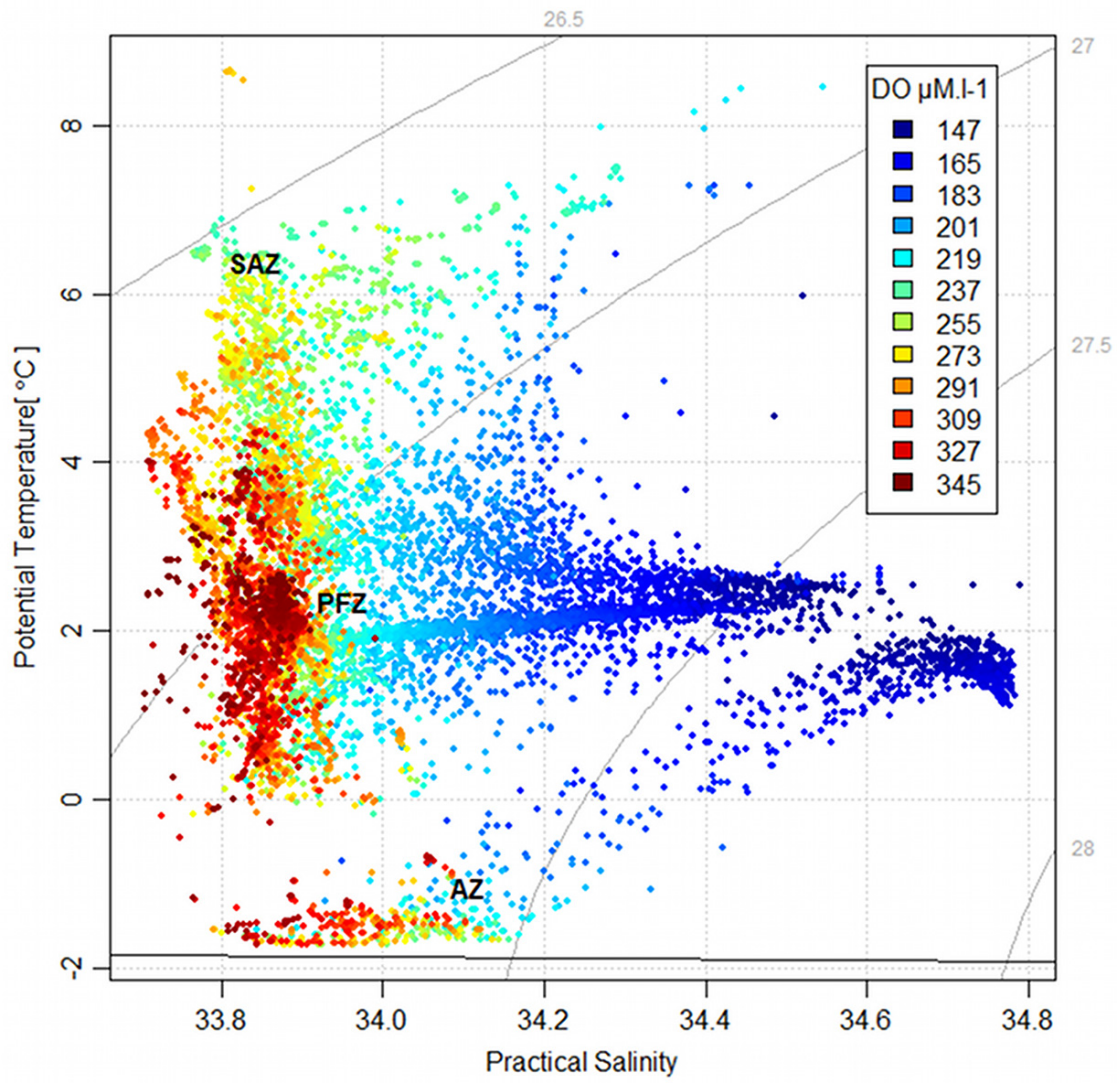
Temperature-Salinity diagram from seal data. Points are coloured by oxygen values. SAZ = Sub-Antarctic Zone, PFZ = Polar Frontal Zone, AZ = Antarctic Zone.

**Fig 6:**
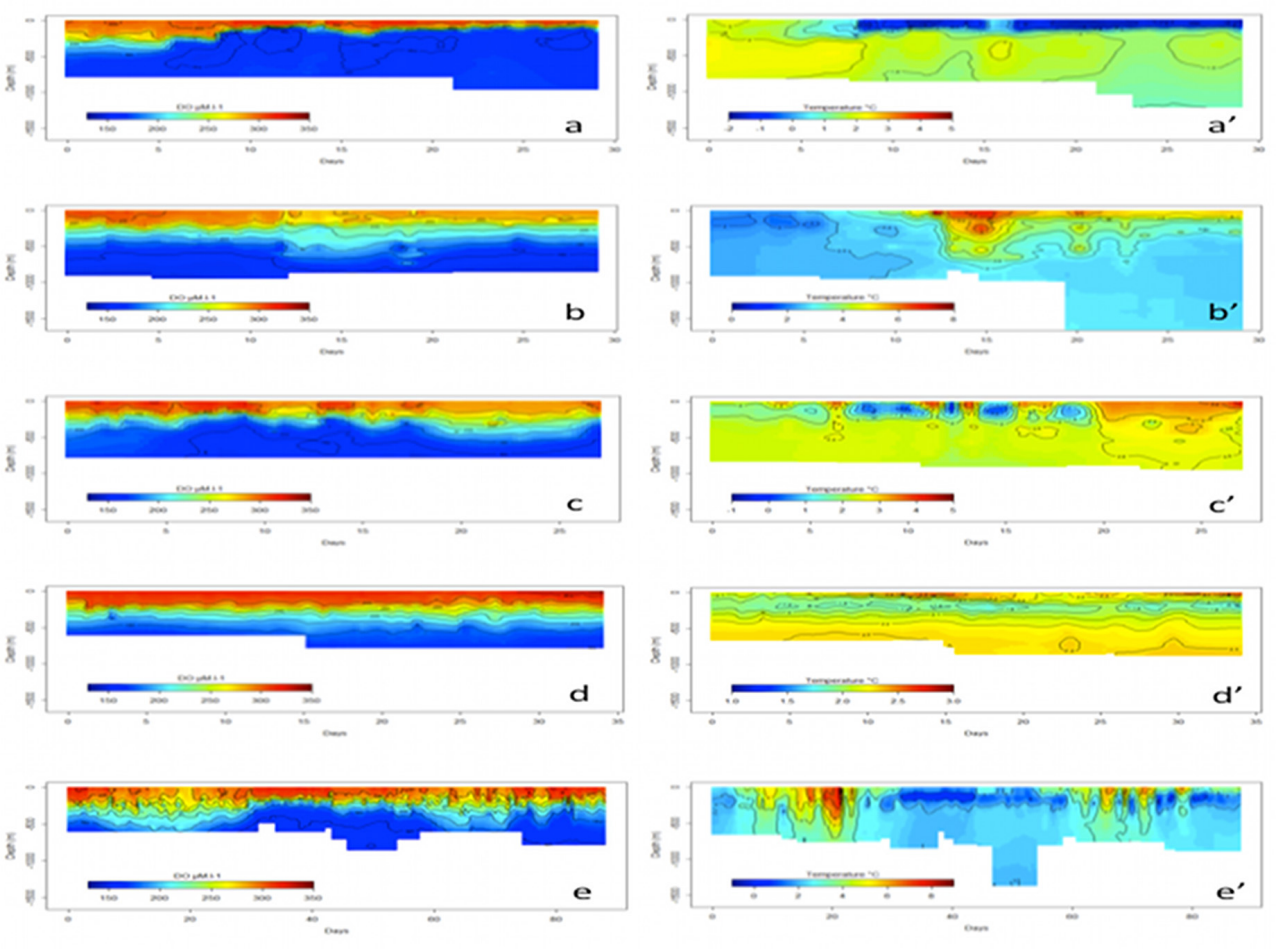
Vertical sections of oxygen and temperature records along each trajectory. Respectively, a) and a’) Individual #1 b) and b’) Individual #2 c) and c’) Individual #3 d) and d’) Individual #4 e) and e’) Individual #5. (See Figs 1, 7 & 9 for the location of each section).

**Fig 7:**
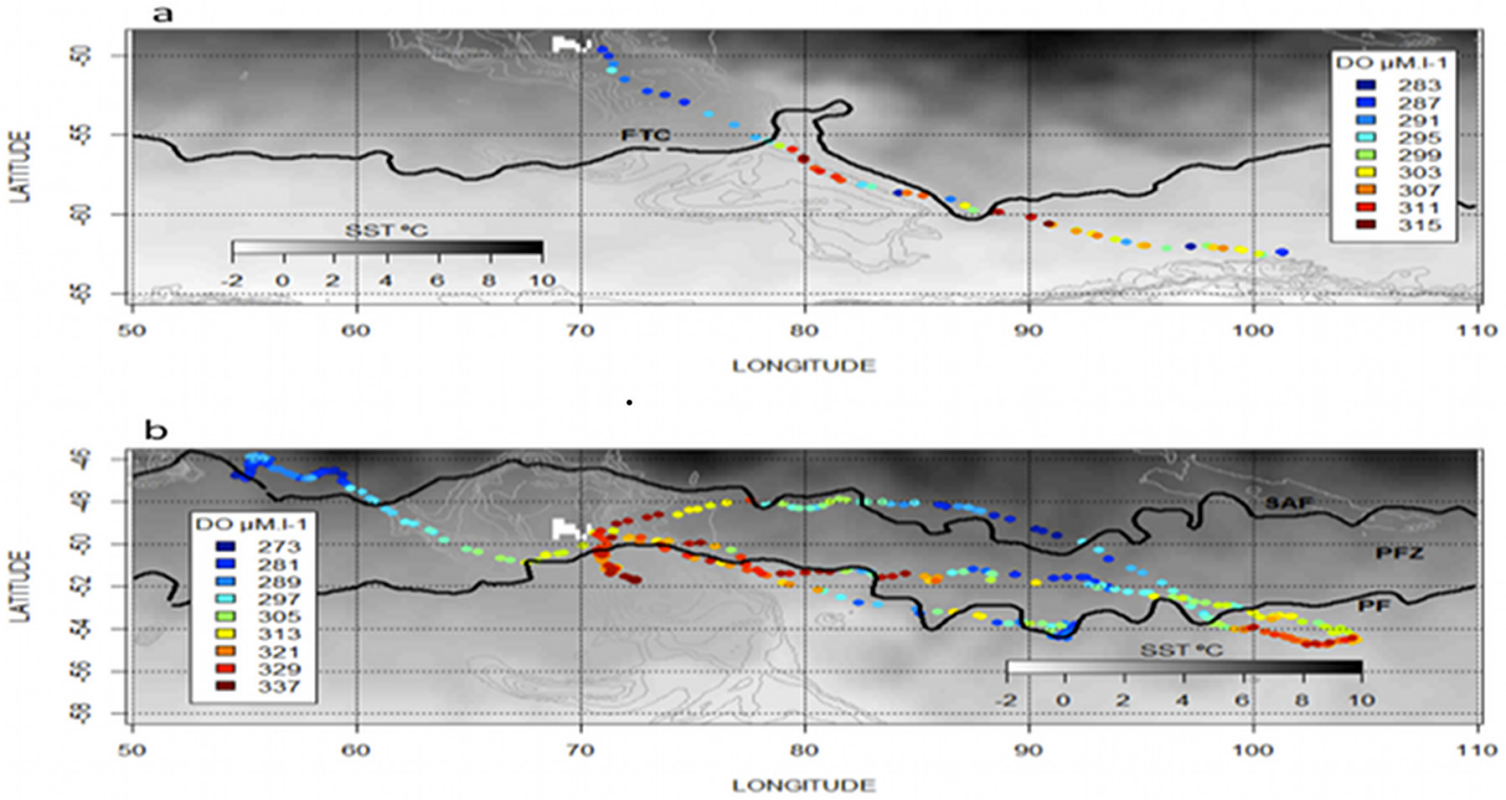
Distribution of surface oxygen in the physical context of the Southern Ocean. a) In 2010, the more oxygenated waters were mainly located south to the Fawn Through Current (FTC), also called the surface expression of the Polar Front and here symbolized by the 0.5°C isotherm. b) In 2011, the surface waters North to the Subantarctic Front (SAF) were low oxygenated compared to surface waters located South to the Polar Front. Within the Polar Frontal Zone (PFZ), no clear pattern appears in the distribution of dissolved oxygen regarding to the main physical structures.

**Fig 8:**
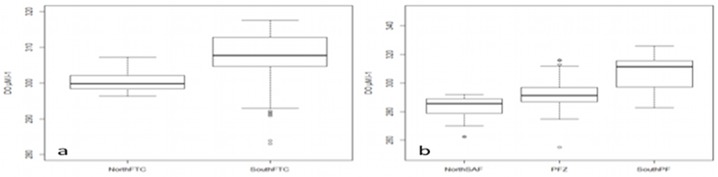
Difference in dissolved oxygen concentration along the seal tracks regarding the position of the main oceanic fronts a) in 2010 b) in 2011. The fronts were defined by the sea surface temperature and the literature.

**Fig 9:**
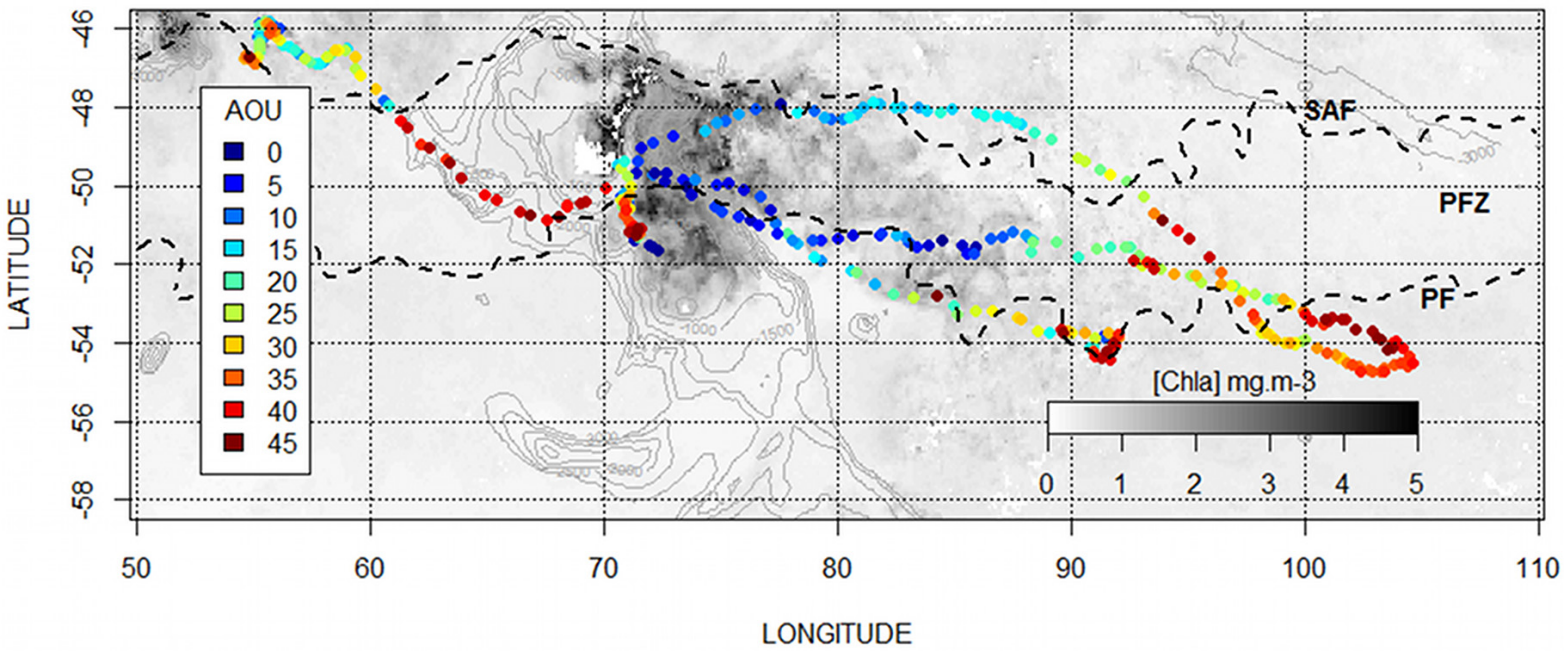
Distribution of surface Apparent Oxygen Utilization in the biological context of the Southern Ocean in 2011. The AOU values indicate that the most oxygenated surface waters (i.e. the lowest AOU) within the PFZ and north to the SAF were related to the higher chlorophyll-a concentrations. No relation was observed in 2010.

**Fig 10:**
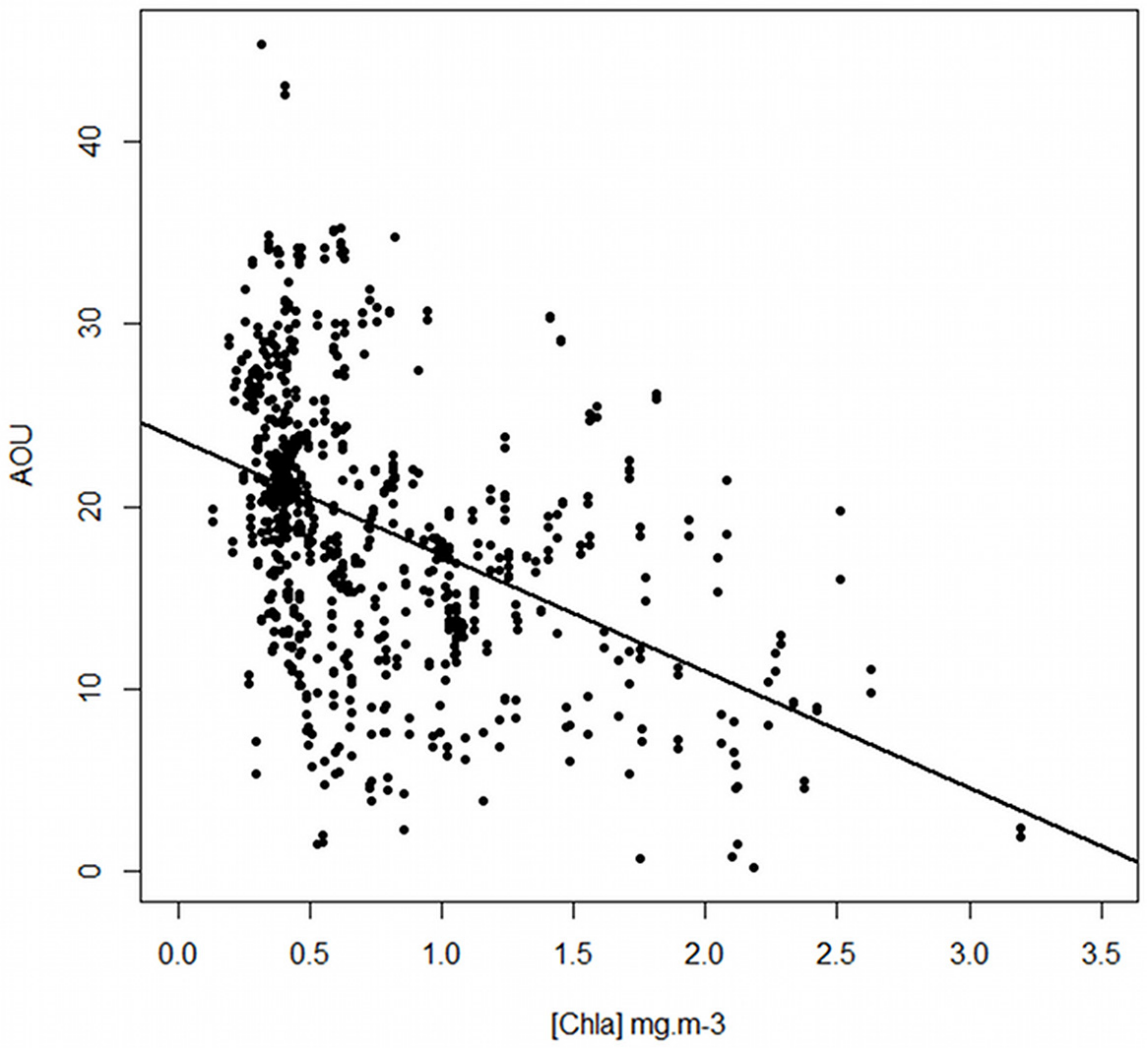
Relationship between the AOU values along the seal tracks and the mean concentration of Chl-a extracted under each location recorded in 2011. The higher the concentration in Chl-a, the lower the values of AOU. The black line represents the significant linear regression: *y* = −6.4*x* + 23.7, p < 0.001.

The distribution of dissolved oxygen according to the water masses was represented on a Temperature-Salinity Diagram, as well as on vertical sections in combination with temperature (Figs 5, 6A–6E and 6A’–6E’). At the sea surface, the distribution of dissolved oxygen was clearly related to the oceanic frontal structures of the SO (Fig 7). In 2010, the most oxygenated surface waters were located South to the 0.5°C isotherm, which symbolize the surface expression of the Polar Front [19], also called the Fawn Trough Current (FTC) [20] (307.0 ± 7.5 μM.l^-1^ South to the front vs. 300.6 ± 3.0 μM.l^-1^ North to the front, *t*_(1,104)_ = 6.38, p < 0.001) (Figs 7A and 8A). In 2011, the surface waters North to the Subantarctic Front (SAF) were low oxygenated (Fig 7B) compared to surface waters located South to the Polar Front (NorthSAF 284.8 ± 13.1 μM.l^-1^; SouthPF 307.1 ± 13.3 μM.l^-1^, *t*_(1,201)_ = 15.37, p < 0.001) (Figs 7B and 8B). Within the Polar Frontal Zone (PFZ) located between the SAF and the PF, no clear pattern appears in the distribution of dissolved oxygen regarding to the main physical structures. However, the use of the Apparent Oxygen Utilization (AOU) indicates that the most oxygenated surface waters (i.e. corresponding to the lowest AOU values) within the PFZ and north to the SAF were related to the higher chlorophyll-a concentration (*F*_(1,738)_ = 169.3, p < 0.001, R^2^ = 0.2) (Figs 9 and 10). No relation between chlorophyll-a and oxygen was observed in 2010.

## Discussion

As temperature, salinity and fluorescence, dissolved oxygen is an important parameter needed to be measured for improving our understanding of the functioning of oceans. Since under-sampling of dissolved oxygen represents the main cause for our limitation in understanding and quantifying key oceanic biological and biogeochemical processes, any new input data is very useful. Moreover, oceanic processes occurring over a continuum of spatial and temporal scales (e.g. mesoscale, “event” scale, seasonal scale), it is of a crucial importance to use a way of measuring, which reflects as closer as possible such a continuum.

Diving almost continuously at great depth during most of the year and covering large distance through remote areas of the Southern Ocean, Kerguelen elephant seals prove to be relevant alternatives to other instruments for monitoring the oceans [9]. The seal-derived measurements of oceanic dissolved oxygen provided both a broader and a finer spatial and temporal resolution than was possible using any other method. Moreover, a number of studies have shown that these animals focus their foraging activity within particular oceanic structures, such as mesoscale eddies [21, 22], whose role in driving the magnitude of biogeochemical fluxes is still largely misunderstood [23, 24]. The seal-derived measurements of dissolved oxygen could contribute to a better understanding of the role of these structures in the global biogeochemical cycles.

However, regardless of the instruments used, dealing with calibration and drift of sensors and controlling quality of data from dissolved oxygen devices remain a major challenge [25, 26]. Although the oxygen concentrations measured by seals are consistent with those obtained from a concomitant ship survey and with previous measurements conducted in close areas [27, 28], the observation of an important temporal drift of the oxygen sensors used in our study as well as a probable offset of measurements in the oceanic mixed layer require the values to be considered cautiously. Why the drift occurred after about 30 days is not clear but a bleaching or a degradation of the sensing foil constitutive of the oxygen sensor or changes in the optical properties of the system over time might be some sources of explanation [29]. The development of a micro-environment around the sensor can also produce oxygen levels that are different from the true ambient conditions [30]. A mechanical cleaning of the sensing foil would be required to solve this issue. Furthermore, the offset of values measured in the mixed layer could be induced by the response time of the thermistor (temperature sensor in the Optode). Thermal diffusion from seawater to the thermistor (inside an aluminium housing) is relatively slow, yielding a lag between the actual temperature of the Optode and the measured Optode temperature. The lag is particularly important in widely varying temperature conditions. In every cases, whatever the origins of mismeasurements, the gaps observed in our study reinforce the absolute necessity to proceed to appropriate calibrations and controls (requiring both laboratory developments and field testing) before any broader utilisation of these data [25], as it has been done in the past for other seal oceanographic parameters [15, 31].

Nevertheless, the relative information on characteristics of water masses with dissolved oxygen provided by seals (i.e. low vs. high oxygenated areas) proves to be promising. Ranging from sub-Antarctic to Antarctic waters through the Southern Ocean, elephant seals look to be efficient assistants for delivering information about the physical and bio-geochemical status of the oceans. They revealed as expected the higher solubility of cold Antarctic waters to oxygen compared to the warmer waters at lower latitudes. They also provided new additional information on the properties of the different water masses they passed through. Such information could be useful to investigate the rate of surface-to-deep ocean circulation and mixing processes [2, 9]. Furthermore, moving east to the Kerguelen Islands, they provided information on the biological source of oxygen induced by the phytoplankton bloom occurring in summer in this area [32]. More than an indication of primary production, these records are of a crucial importance for assessing the air-sea gas-exchange in the Southern Ocean. Beyond the importance of dissolved oxygen to assess the health of the oceans and ultimately the Earth climate, measurements of such a parameter by diving predators help to evaluate the environmental conditions for marine life and open new fields of investigation in the study of marine ecosystems.

Although not directly used by air-breathing predators, such as elephant seals, dissolved oxygen is crucial for life of many other marine organisms, such as potential fish or squid prey of seals, and its direct measurement by predators could provide essential information on the distribution and the habitat of their prey [33]. Previous studies have highlighted a possible relationship between foraging depths of sperm whales and the position of the ocean's oxygen minimum layer through the water column [34]. Monitoring dissolved oxygen directly by marine predators in combination with their foraging behaviour might address this issue. Moreover, dissolved oxygen directly impacts the distribution and the behaviour of other marine predators, such as large fish like swordfish, tuna or sharks [35–37], for which management and conservation measurements could be improved by a better understanding of their habitat. More locally, the extension of coastal oxygen minimum zones (OMZ), which increases and decreases naturally and seasonally, drives the biological activity of marine life and fisheries along the shore of many developing countries [38]. Now, horizontal and vertical expansion of the OMZ is strongly affected by climate change and the depletion in dissolved oxygen related [39]. Marine predators, which evolve in these key ecosystems, represent an opportunity to assess the ecological and socio-economical impacts of ongoing and forthcoming change in dissolved oxygen concentration within these systems.

Given the utility of oxygen for determining consequences of climate changes on marine ecosystems and for a better understanding of ecological interactions in the ocean, recording routinely and continuously its concentration, thanks to predators, proves to be particularly relevant and promising. We recommend that serious attention be given to this approach in many key ecosystems within the world ocean.

## Supporting Information

S1 Dataset This file contains the oxygen raw data used in the analyses. (ZIP)
